# Simple virilizing form of 21-hydroxylase deficiency presenting with renal Insufficiency and polycythemia: a case report

**DOI:** 10.3389/fendo.2026.1759387

**Published:** 2026-02-18

**Authors:** Lu Liang, Xudong Su, Yifan Zhang, Zheng Wang, Guifeng Zhang, Jie Bai, Jian Li

**Affiliations:** 1 Department of Endocrinology, Liaocheng People’s Hospital, Liaocheng, Shandong, China; 2 Institute of Neuropathology, University Hospital Rheinisch-Westfälische Technische Hochschule (RWTH) Aachen, Aachen, Germany

**Keywords:** 21-hydroxylase deficiency, congenital adrenal hyperplasia, hyperandrogenism, renal insufficiency, secondary polycythemia, simple virilizing form

## Abstract

The simple virilizing (SV) form of 21-hydroxylase deficiency (21-OHD) is primarily characterized by androgen excess. Gonadal dysfunction is widely acknowledged; however, systemic complications, including renal injury, are often overlooked. This report presents a rare case involving a 25-year-old female diagnosed with SV 21-OHD, who subsequently experienced unanticipated renal insufficiency and secondary polycythemia. Genetic analysis confirmed compound heterozygous mutations in the *CYP21A2* gene. The patient demonstrated significant hyperandrogenemia and polycythemia driven by erythropoietin. Following erythrocytapheresis and glucocorticoid replacement therapy, androgen levels normalized, resulting in marked renal function recovery and resolution of polycythemia. This case and a mechanistic review illustrate the potential interplay between chronic hyperandrogenism, erythropoiesis dysregulation, and kidney injury, underscoring the importance of timely hormonal management for the preservation of long-term renal function in CAH (Congenital adrenal hyperplasia).

## 1 Introduction

CAH comprises a group of autosomal recessive disorders characterized by impaired steroidogenesis. The most common variant, 21-OHD caused by *CYP21A2* mutations, accounts for over 90% of cases (1). Depending on residual enzymatic activity, 21-OHD is stratified into classical (salt-wasting and SV) and non-classical forms (2).

While the SV subtype typically presents with androgen excess and genital ambiguity, the systemic consequences of chronic hyperandrogenemia beyond the reproductive axis remain underappreciated. Long-term androgen exposure is known to impact metabolic and cardiovascular health (3, 4); however, its potential link to renal injury and erythropoiesis dysregulation is rarely documented. Here, we report a rare case of SV 21-OHD complicated by unexpected renal insufficiency and secondary polycythemia. Through this case and a mechanistic review, we explore the pathogenic interplay between androgen-driven erythrocytosis and renal toxicity, emphasizing the critical need for strict hormonal control to preserve multi-organ function.

## 2 Case presentation

### 2.1 Patient information and clinical findings

A 25-year-old female was admitted to the Nephrology Department with a two-year history of unexplained fatigue and documented elevation in serum creatinine. Her past medical history was notable for atypical genitalia at birth. She was diagnosed with CAH at the age of 7, when she underwent penectomy and external genital reconstruction surgery. Postoperatively, she was treated with oral prednisone (dosage undocumented). Menarche occurred at age 12 but was followed by oligomenorrhea. Due to poor adherence, prednisone was discontinued at age 17, leading to the development of secondary amenorrhea, and she never returned for follow-up. She denied any history of residence at high altitudes or chronic comorbidities, including hypertension, cardiovascular disease, pulmonary disorders, diabetes mellitus, or autoimmune rheumatic diseases. In addition, the patient denied the use of exogenous androgens, anabolic drugs, and nephrotoxic medications.

#### 2.1.1 Clinical findings

Upon admission, the patient’s vital signs were stable, with a body temperature of 36.9 °C, pulse rate of 93 beats/min, respiratory rate of 18 breaths/min, and blood pressure of 126/84 mmHg. Anthropometric measurements indicated obesity, with a weight of 72 kg, height of 150 cm, and a body mass index (BMI) of 32 kg/m². Physical examination was remarkable for significant hyperpigmentation in skin folds, particularly affecting the posterior neck, axillae, and the extensor surfaces of the finger joints. Signs of virilization were prominent, including a deep voice, a robust muscular physique, and hirsutism (observed on the upper lip, chin, lower abdomen, and thighs, with a Ferriman-Gallwey score of 16). Gynecological examination revealed normal female external genitalia post-reconstruction. Systemic examination of the heart, lungs, and abdomen revealed no abnormalities.

### 2.2 Diagnostic assessment

#### 2.2.1 Laboratory and renal evaluation

Initial biochemical assessment revealed stable general metabolic parameters; white blood cell count, platelet count, lipid profile, liver function tests, fasting blood glucose, blood urea nitrogen (BUN), and electrolytes (sodium, potassium) were all within normal reference ranges. However, renal function tests indicated impairment, with a serum creatinine of 100.6 μmol/L (normal range 41-73μmol/L) and elevated blood uric acid (UA) of 484 μmol/L (normal range 155-357μmol/L). Urinalysis demonstrated 1+ proteinuria. Quantitative analysis showed a urine albumin-to-creatinine ratio (UACR) of 146.54 mg/g (normal range 0-30mg/g) and a urine protein-to-creatinine ratio (UPCR) of 263.92 mg/g (normal range 0-150mg/g), with negative urinary red blood cells. The estimated glomerular filtration rate (eGFR), calculated using the CKD-EPI formula (5), was 66.92 ml/min/1.73m², consistent with renal insufficiency.

#### 2.2.2 Immunological and hematological screening

To exclude secondary causes of renal and hematological abnormalities, an extensive serologic workup was performed. The antinuclear antibody (ANA) profile, ANA titer, and serum complement levels (C3/C4) were negative/normal. Viral serologies for hepatitis B surface antigen, hepatitis C antibodies, and HIV were non-reactive. Hematological evaluation revealed significant polycythemia, with a red blood cell (RBC) count of 6.51×10¹²/L (normal range 3.8-5.1×10¹²/L) and hemoglobin (Hb) levels of 208 g/L (normal range 115–150 g/L). Erythropoietin (EPO) level was normal. Primary polycythemia vera and myeloproliferative neoplasms were ruled out based on bone marrow aspiration biopsy, which showed no evidence of primitive or immature cells. Furthermore, genetic testing for JAK2, MPL, BCR/ABL, and CALR mutations yielded negative results.

#### 2.2.3 Endocrine and imaging workup

Hormonal assays revealed a profile characteristic of 21-OHD. Results showed markedly elevated testosterone (9.29 ng/mL, normal range 0.1-0.75 ng/mL), progesterone (>40 ng/mL, normal range 0.31-18.56 ng/mL), 17-hydroxyprogesterone (17-OHP) (456ng/mL, normal range 0.13- 2.41 ng/mL) and adrenocorticotropic hormone (ACTH) (943.5 pg/mL, normal range 10.1-57.6 pg/mL). Conversely, cortisol, luteinizing hormone (LH), and follicle-stimulating hormone (FSH) levels were suppressed. Levels of growth hormone, prolactin and parathyroid hormone remained within normal limits. Imaging studies provided further anatomical evidence. Adrenal computed tomography (CT) demonstrated bilateral thickening of the adrenal glands and a solitary cyst in the right kidney. Gynecological ultrasound indicated a hypoplastic uterus with smaller-than-normal volume. No undescended testes were identified in the inguinal or pelvic regions. Ultrasound examination of the liver, pancreas and spleen, echocardiography, and pituitary MRI were unremarkable.

#### 2.2.4 Genetic confirmation

Karyotype analysis confirmed a 46, XX genotype. To differentiate the functional *CYP21A2* gene from its highly homologous pseudogene *CYP21A1P*, long-range PCR amplification followed by next-generation sequencing (NGS) was performed on genomic DNA extracted from peripheral blood leukocytes. The analysis identified compound heterozygous mutations in the *CYP21A2* gene, both classified as Pathogenic according to ACMG guidelines (6):

1. A missense mutation c.1069C>T (p.Arg357Trp) located in Exon 8, inherited from the father.
2. A splice-site mutation c.293-13C>G (p.)? located in Intron 2, inherited from the mother. This variant is known to disrupt normal splicing by activating a cryptic splice site, leading to a 19-bp insertion, a subsequent frameshift, and the production of a truncated protein with total loss of enzymatic activity (7).

**Figure 1:**
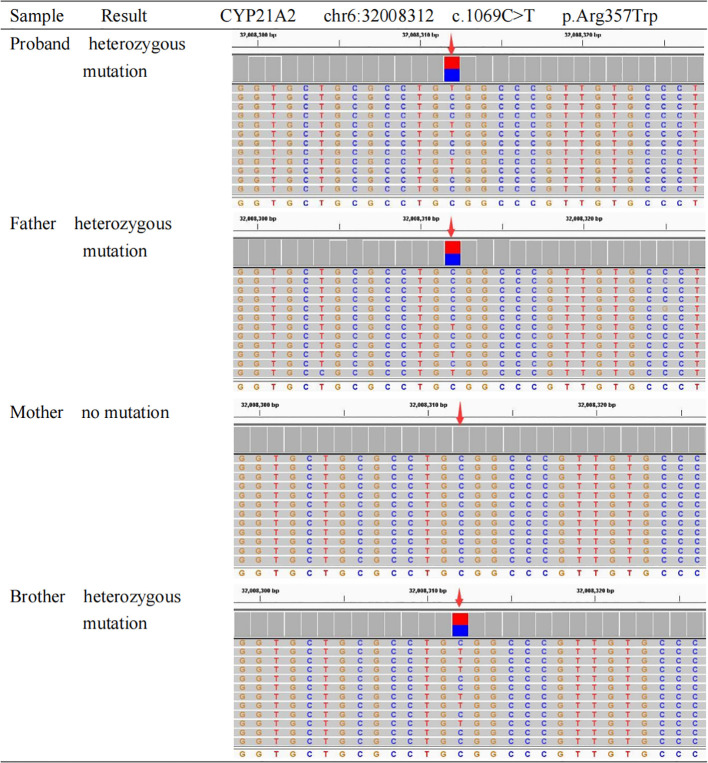
Sanger sequencing analysis of the CYP21A2 c.1069C>T mutation in the family. The chromatograms illustrate the DNA sequence of Exon 8. The red arrows indicate the nucleotide position c.1069. The sequencing results reveal that the proband (patient), father, and brother are heterozygous for the C>T transition (indicated by double peaks), confirming the presence of the c.1069C>T (p.Arg357Trp) mutation. The mother exhibits a wild-type genotype (C/C) at this locus. This confirms the paternal origin of this mutation.

**Figure 2:**
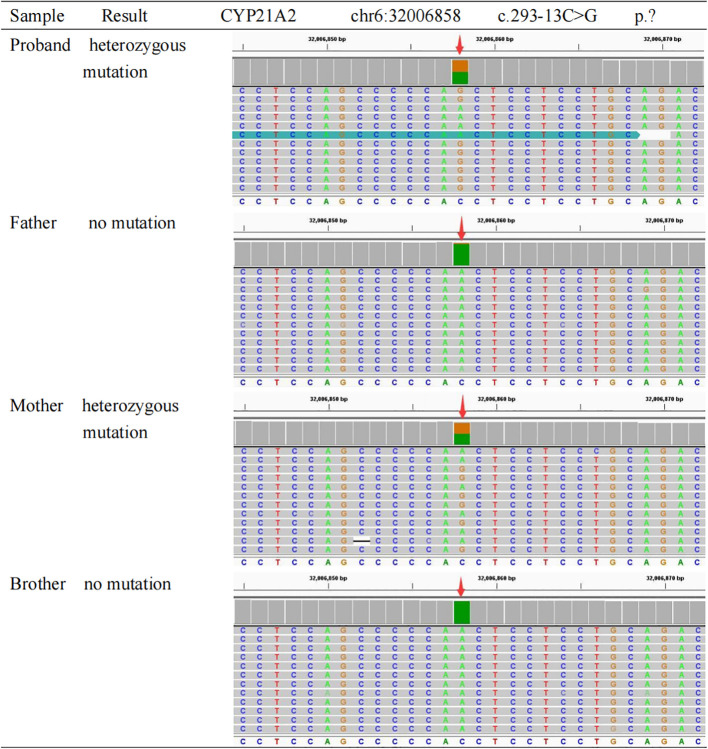
Sanger sequencing analysis of the CYP21A2 c.293-13C>G mutation in the family. The chromatograms display the DNA sequence of Intron 2. The red arrows indicate the splice-site position c.293-13. The sequencing results demonstrate that the proband and mother are heterozygous for the C>G transversion (indicated by double peaks), confirming the presence of the c.293-13C>G (p.)? splice-site mutation. The father and brother exhibit a wild-type genotype (C/C) at this locus. This confirms the maternal origin of this mutation.

Sanger sequencing validated these findings and confirmed the familial segregation (**Figures 1**, **2**): the father was heterozygous for c.1069C>T, the mother was heterozygous for c.293-13C>G, and the patient’s younger brother was identified as a carrier of the paternal mutation (c.1069C>T) but negative for the maternal mutation. Additionally, Multiplex Ligation-dependent Probe Amplification (MLPA) analysis excluded large gene deletions or duplications. These molecular findings provided a definitive diagnosis of SV 21-OHD.

### 2.4 Therapeutic intervention and outcomes

To manage the severe polycythemia and underlying hormonal imbalance identified at baseline (**Table 1**), the patient initially underwent erythrocytapheresis and was initiated on Dexamethasone 0.75 mg daily. At the two-month follow-up, the suppression of the hypothalamic-pituitary-adrenal (HPA) axis was evident, with testosterone levels dropping slightly below the reference range. Consequently, the regimen was transitioned to oral Prednisone 5 mg daily. However, by the three-month follow-up, a significant clinical rebound occurred: ACTH surged to 645.4 pg/mL and testosterone rose to 2.65 ng/mL, indicating insufficient control by prednisone.

**Table 1:** Biochemical and endocrine parameters at baseline.

Biochemical parameters	Before hemapheresis	After hemapheresis	Normal range
RBC (×10^^12^/L)	6.51	4.79	3.8-5.1
Hb (g/L)	208	155	115-150
Cr (umol/L)	100.3	88.4	41-73
BUN (mmol/L)	5.07	4.57	2.6-7.5
UA (umol/L)	505	412	155-357
sodium (mmol/L)	137	137.9	137-147
potassium (mmol/L)	3.95	3.87	3.5-5.3
Proteinuria quantification	+		
UACR (mg/g)	146.54	–	0-30
UPCR (mg/g)	263.92	–	0-150
24-hour urine protein (mg/24h)	239.08		0-150
Endocrine parameters			
ACTH (pg/mL) 8am	943.5	–	10.1-57.6
4pm	85.38	–	–
0am	60.03	–	–
Cortisol (ug/dL) 8am	5.35	–	6.7-22.6
4pm	5.37	–	0-10
0am	1.7	–	–
17-OHP (ng/mL)	456		0.13-2.41
Progesterone (ng/mL)	>40	–	0.31-18.56
LH (mIU/mL)	0.2	–	1.2-103.03
FSH (mIU/mL)	0.52	–	1.79-22.51
Estradiol (pg/mL)	53.99	–	15.16-442.62
Testosterone (ng/mL)	9.29	–	0.1-0.75

**Figure 3:**
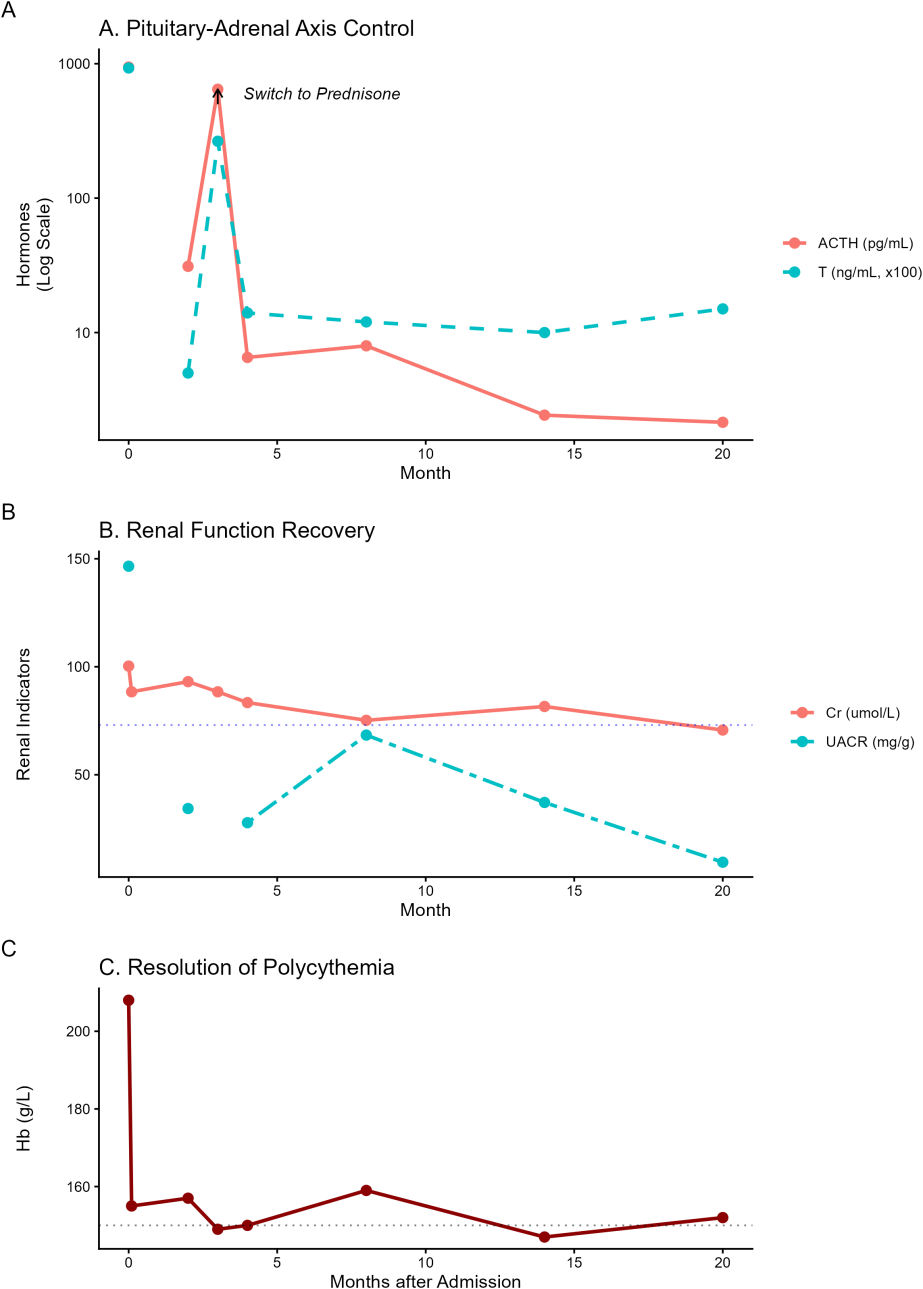
Dynamic trends of endocrine, renal, and hematological parameters over 20 months. **(A)** Pituitary-Adrenal Axis Control: Serum ACTH and testosterone levels (logarithmic scale) showing rapid suppression post-treatment. Note the transient rebound at Month 3 (arrow) caused by a medication switch from dexamethasone to prednisone, followed by stabilization after reverting to dexamethasone. **(B)** Renal Function Recovery: Longitudinal changes in serum creatinine (Cr) and UACR. Renal recovery closely paralleled the control of adrenal androgens, with both markers normalizing by the 20-month follow-up (dotted line: Cr upper limit of normal). **(C)** Resolution of Polycythemia: Hemoglobin (Hb) levels remained stable below 150 g/L (dotted line) throughout the follow-up period, following initial erythrocytapheresis and sustained hormonal suppression.

The therapy was subsequently optimized by reverting to Dexamethasone at a reduced dosage of 0.375 mg daily. This adjustment elicited a robust and sustained clinical response. Longitudinal follow-up (**Figure 3**) revealed a synchronized recovery of multi-organ functions compared to the initial baseline state. As illustrated in **Figure 3A**, the transient switch to prednisone at Month 3 caused a distinct spike in androgen activity, which was promptly suppressed upon the resumption of low-dose dexamethasone.

Parallel to these hormonal shifts, renal function showed marked and steady improvement (**Figure 3B**) from the initial insufficiency reported in **Table 1**. By the 20-month follow-up, serum creatinine had decreased to 70.7 μmol/L (reference range: 41–73 μmol/L), and the eGFR recovered to 101.8 mL/min/1.73m². The UACR also normalized to 9.5 mg/g, with clinical proteinuria becoming negative. Furthermore, secondary polycythemia remained resolved (**Figure 3C**); hemoglobin levels were consistently maintained below 150 g/L after the initial treatment, showing a significant reduction from the baseline value of 208 g/L (**Table 1**). These findings, summarized in **Table 2**, confirm the sustained efficacy of personalized low-dose dexamethasone in managing systemic complications and achieving long-term biochemical stability.

**Table 2:** Biochemical and endocrine parameters at follow-up.

Biochemical parameters	2months	3months	4months	8months	14months	20months	Normal range
RBC (×10^^12^/L)	4.75	4.6	–	4.89	4.44	4.71	3.5-9.5
Hb (g/L)	157	149	–	159	147	152	115-150
Cr (umol/L)	93.1	88.4	83.4	75.2	81.6	70.7	41-73
BUN (mmol/L)	6.24	5.87	4.08	3.58	5.47	3.63	2.6-7.5
sodium (mmol/L)	138.2	–	137.4	137.5	139.9	138.6	137-147
potassium(mmol/L)	4.49	–	4.72	4.38	4.38	4.78	3.5-5.3
Proteinuria quantification	trace	–	trace	trace	trace	negative	Negative
UACR (mg/g)	34.35	–	27.8	68.36	37.17	9.5	0-30
Endocrine parameters							
ACTH (pg/mL) 8am	31.08	645.4	6.53	7.97	2.43	2.15	10.1-57.6
Cortisol (ug/dL) 8am	0.04	2.68	0	0.04	0	0.1	6.7-22.6
17-OHP (ng/mL)	1.5	358	1.6	–	–	–	0.13-2.41
Progesterone (ng/mL)	7.31	>40	5.29	8.28	9.1	13.12	0.31-18.56
LH (mIU/mL)	18.2	0.65	6.52	–	7.85	13.03	1.2-103.03
FSH(mIU/mL)	5.68	0.86	5.13	–	5.48	4.76	1.79-22.51
Estradiol (pg/mL)	30.16	46.79	56.78	–	76.64	43.81	15.16-442.62
Testosterone (ng/mL)	0.05	2.65	0.14	0.12	0.1	0.15	0.1-0.75

## 3 Discussion

### 3.1 Pathophysiology and systemic impact

21-OHD is fundamentally characterized by impaired cortisol biosynthesis, which triggers compensatory ACTH hypersecretion via the HPA axis (8). This drive results in adrenal hyperplasia, the accumulation of steroid precursors (e.g., 17-OHP), and varying degrees of hyperandrogenemia. The SV form, associated with 2-10% residual 21-hydroxylase activity, typically presents with androgen excess—manifesting as virilization in females and precocious puberty in males—but usually spares patients from the life-threatening electrolyte disturbances seen in salt-wasting subtypes (9). However, while gonadal sequelae are well-documented, the systemic impact of chronic hyperandrogenemia on non-reproductive organs remains underappreciated.

### 3.2 Mechanisms of polycythemia

Polycythemia, a rare clinical feature of CAH, was initially described in salt-wasting neonates and later in two adults with SV form. These three cases, including ours, shared the feature of chronic undertreatment, with markedly elevated androgen levels despite normal EPO (10–12). The marked elevation in RBC count and hemoglobin was driven by chronic hyperandrogenemia. Androgens stimulate erythropoiesis through multiple synergistic pathways: they directly upregulate erythropoietin (EPO) synthesis, enhance insulin-like growth factor-1 (IGF-1) activity, and facilitate iron incorporation into erythrocytes (13). In this patient, the EPO level was normal, suggesting that testosterone may increase the biological activity of erythropoietin without affecting its concentration (14). Previous studies have demonstrated a strong positive correlation between testosterone and hemoglobin levels, particularly in women with hyperandrogenic disorders (15). In our case, the normalization of hematological parameters following glucocorticoid therapy provides robust clinical evidence that the polycythemia was secondary to the hormonal imbalance.

The clinical implications of this secondary polycythemia are significant. Increased blood viscosity compromises microcirculation, predisposing patients to thrombotic events and organ ischemia. This risk was underscored by a reported case of a male patient with untreated CAH who suffered a stroke due to polycythemia (11). Similarly, in our patient, we postulate that hyperviscosity-induced renal hypoperfusion acted as a precipitating factor for renal dysfunction.

Polycythemia contributes to hyperuricemia via accelerated purine metabolism. The relationship between testosterone levels and uric acid levels remains controversial. Some studies have found that serum uric acid levels in adult males may be negatively correlated with serum testosterone levels (16). However, in a study of female-to-male transgender individuals with gender dysphoria, testosterone administration was associated with increased serum uric acid levels in a dose-dependent manner (17). This case corresponds to the latter mechanism, wherein androgen excess raises uric acid levels directly by accelerating purine catabolism and indirectly by promoting insulin resistance and central obesity.

### 3.3 Renal injury: a novel association

There was a reported case of a newborn diagnosed with classic 21-OHD, who presented with acute renal insufficiency and hematuria. These manifestations were associated with factors such as dehydration, hyponatremia, and deep vein thrombosis (18). To our knowledge, chronic renal insufficiency has not been reported as a complication of 21-OHD. However, literature from sports medicine documents renal injury in bodybuilders using anabolic steroids, including focal segmental glomerulosclerosis (FSGS) and tubular atrophy (19). The temporal synchronization observed in our patient between androgen suppression and renal recovery (**Figures 3A, B**) provides strong clinical evidence for this mechanistic link. As the excessive testosterone was neutralized through optimized glucocorticoid therapy, the hemodynamic stress and metabolic burden on the kidneys appeared to alleviate concurrently. This is evidenced by the steady normalization of serum creatinine and the significant reduction in UACR over the 20-month follow-up period, reinforcing the hypothesis that hyperandrogenism acts as a reversible driver of renal impairment in female 21-OHD patients. We propose that the renal injury in this case resulted from a “triple-hit” mechanism:

1. Hemodynamic Stress: Polycythemia increased blood viscosity, leading to chronic renal microcirculatory ischemia (20).
2. Metabolic Toxicity: Hyperuricemia, a consequence of high cell turnover, likely exacerbated tubular injury through urate crystal deposition and inflammation.
3. Direct Androgen Cytotoxicity: Testosterone exerts direct deleterious effects on renal tissue. In Adriamycin-induced nephropathy models (21), male rats exhibit more severe glomerulosclerosis than females, and exogenous testosterone accelerates mesangial sclerosis. Molecularly, testosterone activates podocyte androgen receptors to trigger apoptosis independent of TGF-β1 and amplifies renal inflammation via TNF-α upregulation (22). Furthermore, androgens stimulate the renin-angiotensin-aldosterone system (RAAS), promoting profibrotic remodeling and hypertension (23).

### 3.4 Role of obesity and differential diagnosis

Obesity is a recognized risk factor for glomerulopathy. While the patient’s BMI of 32 kg/m² could potentially contribute to renal stress, obesity-related nephropathy typically follows a slow, progressive course (24). In contrast, our patient exhibited a rapid and significant recovery of renal function (eGFR improved from 66.9 to 89.7 ml/min/1.73m²) and resolution of proteinuria within three months of hormonal control. Within three months of hormonal control. As illustrated in the long-term trend (**Figure 3B**), this recovery was not transient but sustained, with serum creatinine ultimately reaching a stable nadir of 70.7 μmol/L by Month 20. This sustained reversibility further distinguishes the androgen-mediated toxicity from the typically slow and progressive course of obesity-related glomerulopathy. This reversibility strongly suggests that testosterone-mediated toxicity, rather than obesity, was the primary driver of renal impairment, mirroring the reversible renal injury observed in bodybuilders upon steroid cessation (19). Based on the improvement in renal parameters, the patient declined renal biopsy. Nevertheless, the potential impact of obesity-related nephropathy warrants further investigation, particularly in patients with congenital adrenal hyperplasia who may harbor additional risk factors for chronic kidney disease. The patient’s refusal of a renal biopsy following clinical improvement represents a key study limitation, which prevented definitive histopathological classification.

Nephrocalcinosis is a rare complication of CAH, often accompanied by hypercalcemia and hypercalciuria, which can impact renal function. Studies have confirmed a higher incidence in male children. The patient’s age, gender, normal blood calcium levels, and absence of renal calcification deposits do not support a diagnosis of nephrocalcinosis (25, 26).

### 3.5 Delayed diagnosis and newborn screening

CAH is a relatively common genetic disorder, for which glucocorticoid therapy serves as an effective treatment option. In cases of salt-wasting 21-OHD, early diagnosis significantly reduces the risk of adrenal crisis. For patients with the SV form, early diagnosis helps prevent misassignment of female external genitalia, avoid psychological trauma associated with atypical sexual development, and allow timely initiation of glucocorticoid therapy, thereby effectively suppressing adverse outcomes caused by hyperandrogenism, such as accelerated growth and advanced bone age. Some countries recommend newborn screening for CAH. However, there is some controversy regarding the screening for the SV form. The potential benefits of early intervention for asymptomatic mild hyperandrogenemia remain uncertain, whereas long-term glucocorticoid treatment may pose metabolic and cardiovascular risks. As many individuals with the SV form of CAH have only mild adult symptoms, neonatal screening could lead to their over-medicalization. In resource-limited settings, incorporating the SV form into CAH screening may be cost-effective. Ethically, however, mandatory screening for this non-life-threatening variant is controversial, as it may infringe upon an individual’s future autonomy.

### 3.6 Therapeutic implications and glucocorticoid selection

The clinical significance of personalized glucocorticoid titration is vividly illustrated by the ‘rebound phenomenon’ captured at Month 3 (**Figure 3A**). In our case, the transient switch from dexamethasone to prednisone resulted in an immediate escape of ACTH and testosterone from hormonal control, which was only reversed upon reinstating low-dose dexamethasone (0.375 mg daily). This fluctuation, clearly visualized in the longitudinal trend, underscores that for certain SV 21-OHD patients with extreme hyperandrogenemia and systemic complications, conventional prednisone doses may lack the necessary potency to stabilize the HPA axis. The superior suppressive effect of dexamethasone—when carefully titrated—offers a critical therapeutic window to achieve biochemical stability and prevent the recurrence of renal hemodynamic stress and polycythemia.

## 4 Conclusion

This report documents the rare co-occurrence of polycythemia and renal insufficiency in the SV form of 21-OHD. The pathology likely involves a complex interplay of testosterone-driven erythropoiesis, microcirculatory impairment, and direct renal toxicity. Treatment with erythrocytapheresis and glucocorticoid replacement successfully reversed both hematological and renal abnormalities. This case highlights the critical need for early intervention and rigorous long-term monitoring to prevent systemic organ damage in CAH patients.

## Data Availability

The raw data supporting the conclusions of this article will be made available by the authors, without undue reservation.
